# Cerebro-Spinal Meningitis Occurring during the Prevalence of Epidemic Catarrhal Fever

**Published:** 1849-01

**Authors:** R. L. Scruggs


*Cerebro-Spinal Meningitis occurring during the prevalence of Epidemic Catarrhal Fever.* By R. L. Scruggs, M. D.

Previously to saying anything about the cerebro-spinal affection, I will endeavor to give a brief account of the epidemic catarrhal fever which made its appearance here last fall, while a few cases of the epidemic typhoid fever yet lingered; following close upon the heels of the latter epidemic. Although this epidemic, resembles in its general features the same disease described by various authors, as occurring at different and distant periods, yet it may present some features peculiar and characteristic. Whether this be true or not, it will not, I presume, be uninteresting to notice something of its history, phenomena, complications, &c., and ascertain, if we can, its connections with other morbid conditions of the body, and possibly prove their identity; or show that affections hitherto treated of as separate and distinct diseases, if not the same, are produced by the same remote cause. I think that a fair opportunity of observing one epidemic, and a careful study of all its phases, should go far to convince any one that many cases, apparently dissimilar, from the descriptions given of them, are the same disease, or at least produced by the same morbific agency—modified only by the circumstances attending each epidemic—as climate, season, locality, &c., or each particular case, as constitution, idiosyncrasy, &c. Thus we may have the morbific agent small in quantity, spending its force upon the bronchial or Schneiderian mucous membranes, constituting slight catarrh or bronchitis, which is its most common form; or it may spend its principal force upon the lungs, less frequently upon the stomach and bowels; or lastly, and worse than all, upon the gray matter in the centre of the spinal cord, "the nervous system of reflex actions," producing violent spasmodic action; or it may extend itself to the brain and its coverings, producing intense pain, wild delirium, coma, apoplexy and paralysis.

These and several strange phenomena have presented themselves to me in treating the epidemic that has prevailed here this year, called "influenza, la grippe, spotted fever, cold plague, typhoid pneumonia, cerebro-spinal meningitis, catarrhal and catarrho-rheumatic fever." These names, I think, have been given to the same disease, for all the symptoms attending the above named diseases I have witnessed in this epidemic, except the spotted appearances described by some. Thus I have seen a patient attacked with apparently ordinary cold, as the phrase is; soon soreness of the muscles and intense pain in the joints supervene; great prostration of the vital energies, intense bronchitis, the sputa slightly tinged with blood, and finally death ensuing, apparently from the lungs being filled with tenacious mucous, which the patient is unable to throw up; but before death takes place the same contraction of the muscles of the back is observed as in the other cases. Again, such is the violence of the attack sometimes, that nothing is observed but the cerebro-spinal affection, which often produces death in from six to ten hours.

Manifesting itself in this way it is truly a frightful disease, and such is its protean character, that at times it is extremely difficult, if not impossible, to recognize it. The patient will sometimes complain of intense pain in a circumscribed spot in some part of the body or extremities, as the toe, ankle, wrist, &c., and in these cases it is often death ab initio.

The first violent case of this disease that I was called to see was that of a young man, æt. 20. He was taken with soreness of the muscles the day before, with wandering pains about the chest, and slight bronchitis. He died before I had time to examine him. His whole body was curved strongly forward, the thighs flexed upon the abdomen, so that the forehead rested upon or between the knees. This case proved fatal in twenty-four hours.

My next case was a young lady, æt. 18. The position of her body was exactly the reverse of the first patient; her head and neck were drawn backwards and rigidly fixed, countenance pale and somewhat distorted, respiration slow and gasping, extremities cold, and no pulse at the wrist. This was a formidable array of symptoms, but did not deter me from making an effort to save her, which it probably would have done, had I known more of the character of the disease. I commenced the treatment by scarifications and cups to the back of the head and neck and along the spine, at the same time using active frictions to the extremities with the most stimulating articles that could be procured. A stimulating enema was then ordered, and hot corn and bricks to be placed under the bed clothing, so that the heat could be conveyed to all parts of the body and limbs. The enema seemed to arouse her considerably, and I directed it to be repeated several times. As soon as she could be made to swallow, I gave her a mercurial cathartic, which operated very well in the usual time. Nothing remarkable in the smell or colour of the passages. Early the next morning I visited her, and found her so much improved that I had considerable hopes of her recovery. Warned, however, of the tendency of the disease to return with increased violence, I introduced, as rapidly as possible, quinine, camphor, and Cayenne pepper; but alas, for my hopes! in two or three hours it was but too perceptible that all my efforts would prove in vain. Her countenance assumed a frightened and beseeching aspect; the muscles of the back commenced contracting; soon she became utterly insensible to all surrounding objects, and died in about three hours—her head nearly touching the lower part of her body,—the spinal column, thus bent backwards, forming almost a complete circle.

The next case was that of a young man, æt. 22. He sent for me about 10 o'clock at night, complaining of intense, excruciating pain in the head. He said it felt as though a pointed instrument was pierced through from one temple to the other. The pulse was normal;—cups to the temples and behind the ears, a stimulating foot bath, a dose of hydr. c. creta and Dover's powders, with a little warm infusion of balm, sufficed to relieve the pain entirely, and he slept comfortably during the remainder of the night. When I called to see him the next morning, I was surprised to find him in the act of mounting his horse. I remonstrated against his going out for a day or two; told him that I thought his disease was the prevailing epidemic, with a decided tendency to the brain and spinal marrow; that a relapse would be severe, and might prove fatal. In spite of my warnings, however, he rode six or eight miles, and was caught out in a heavy fall of rain; in twenty hours after which, the disease returned with increased violence, and marched steadily and rapidly on to a fatal termination, in spite of lancet, cups, sinapisms, blisters, and every thing else that could be suggested by myself and a consulting physician, whom I called in to my assistance. In this case there was furious delirium towards the last, but none of the muscles were contracted, except those of the eye, which drew the organ around in such a manner as almost to conceal the iris, which, with the wild delirium, gave to the countenance a singularly distorted and fearful aspect.

The next case that I shall notice was that of a negro woman, æt. 20. She was insensible when I first saw her; breathing stertorously: head intensely hot; pulse 130 beats to the minute, and tolerably full. V. S. ℥xx.; cups to the temples, behind the ears, and down the spine; hot pediluvia and cold water to the head. This produced some amelioration of the symptoms, and I gave her calomel, nitr. potas. and ipecac. every two hours for four times, with spts. nitr. dulc. and vin. ipecac. between. But the next day, notwithstanding the bowels had been well evacuated, I found the pulse more frequent, the head hot and *drawn backwards*, and, indeed, every symptom unfavourable. The cups to the back of the head and down the spine were again resorted to, with cold water to the head, and general tepid bath, after which blisters to the nape of neck and to the extremities; but nothing seemed to afford relief. The bowels were freely acted upon with calomel and ipecac., followed by infusion of senna when required; and this was continued pro re nata. She died on the third day, her head drawn tightly backwards, inclining a little to one side, and paralysis of the right side.

These four cases will suffice to show, in some degree, the violence and protean character of the disease, although it is not a tithe of what might be written upon the subject. If I am right in regarding all the cases that I have treated as the same disease, modified only by different circumstances in different individuals, then the disease cannot be said to be a fatal one; for the four fatal cases, just described, occurred at long intervals, between each of which I treated a large number of cases, without losing one. But regarding the spinal affection as a distinct disease, and the case is altogether different. Some accounts of the disease say that five-sixths die. I must candidly confess that I have never seen a case recover; that is, where the spinal affection has been the prominent symptom: nor have I met with a single physician who has been more successful than myself. One physician informed me that he had effected a cure in one case, he thought, by opening both temporal arteries; and another, that in cupping the temples he accidentally cut the artery, which bled freely, and the patient recovered.

